# l-Alanine activates hepatic AMP-activated protein kinase and modulates systemic glucose metabolism

**DOI:** 10.1016/j.molmet.2018.08.002

**Published:** 2018-08-11

**Authors:** Yusuke Adachi, Ana Luisa De Sousa-Coelho, Ikue Harata, Charlie Aoun, Sandra Weimer, Xu Shi, Karina N. Gonzalez Herrera, Hirokazu Takahashi, Chris Doherty, Yasushi Noguchi, Laurie J. Goodyear, Marcia C. Haigis, Robert E. Gerszten, Mary-Elizabeth Patti

**Affiliations:** 1 Research Division, Joslin Diabetes Center, and Harvard Medical School, Boston, MA, USA; 2 Institute for Innovation, Ajinomoto Co. Inc., Japan; 3 Department of Cell Biology and Paul F. Glenn Laboratories for the Biological Mechanisms of Aging, Harvard Medical School, Boston, MA, USA; 4 Department of Medicine, Beth Israel Deaconess Medical Center, Harvard Medical School, Boston, MA, USA

**Keywords:** Amino acids, Alanine, AMPK, Energy sensor

## Abstract

**Objective:**

AMP activated protein kinase (AMPK) is recognized as an important nutrient sensor contributing to regulation of cellular, tissue, and systemic metabolism. We aimed to identify specific amino acids which could modulate AMPK and determine effects on cellular and systemic metabolism.

**Methods:**

We performed an unbiased amino acid screen to identify activators of AMPK. Detailed analysis of cellular signaling and metabolism was performed in cultured hepatoma cells, and *in vivo* glucose metabolism and metabolomic patterns were assessed in both chow-fed mice and mice made obese by high-fat diet feeding.

**Results:**

Alanine acutely activates AMP kinase in both cultured hepatic cells and in liver from mice treated *in vivo* with Ala. Oral alanine administration improves systemic glucose tolerance in both chow and high fat diet fed mice, with reduced efficacy of Ala in mice with reduced AMPK activity. Our data indicate that Ala activation of AMPK is mediated by intracellular Ala metabolism, which reduces TCA cycle metabolites, increases AMP/ATP ratio, and activates NH_3_ generation.

**Conclusions:**

Ala may serve as a distinct amino acid energy sensor, providing a positive signal to activate the beneficial AMPK signaling pathway.

## Highlights

• Unbiased amino acid screen identified alanine as a unique activator of AMP kinase.
• Alanine acutely activates AMP kinase in both cultured cells and *in vivo*.
• Alanine and NH_3_ metabolism contribute to regulation of AMP kinase activation.
• Effects of Ala are reduced in absence of AMP kinase.
• Oral alanine improves glucose tolerance *in vivo* in both chow and HFD-fed mice.

## Graphical abstract

**Figure:**
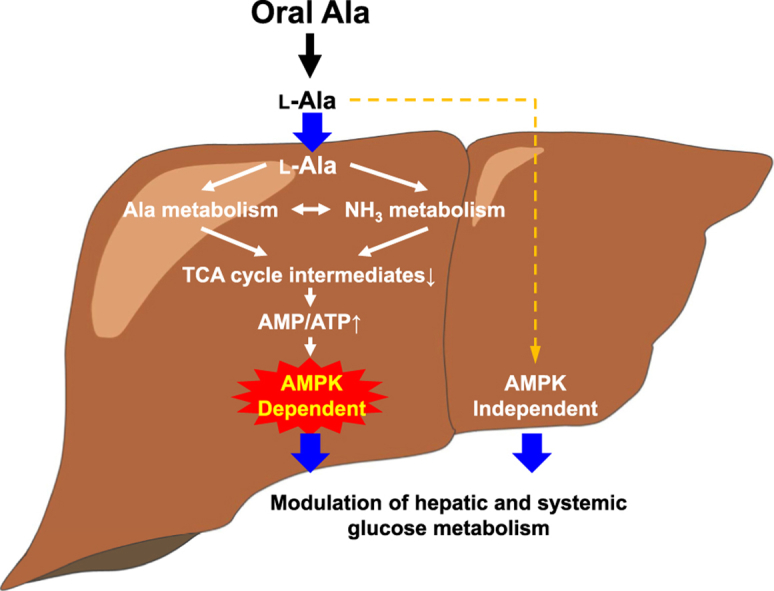

## 1 Introduction

Plasma amino acids have long been recognized to be dysregulated in obesity [1]. The advent of unbiased metabolomics has further strengthened associations between plasma amino acids, metabolic disorders, and type 2 diabetes (T2D) [2], [3], [4], [5], [6], [7], [8], [9]. Furthermore, longitudinal prospective cohorts indicate that a combination of branched chain and aromatic amino acids can predict T2D [10] and are associated with progression of insulin resistance in children [5]. While increased amino acids, particularly branched chain amino acids (BCAA), may reflect inadequate insulin action [1] and unrestrained proteolysis or inadequate metabolism [11], [12], [13], studies in cultured cells and animals [14], [15] have also indicated that amino acids can directly modulate insulin action and may contribute to the pathophysiology of insulin resistance. However, the molecular mechanisms responsible for these effects remain uncertain.

BCAA can activate the anabolic mTOR pathway [16], [17]. Amino acids can also modulate the nutrient sensor AMP kinase (AMPK) [18], a serine-threonine kinase activated by energetic stress (increased AMP/ADP), hypoxia, and other signaling pathways. AMPK inhibits mTOR signaling and stimulates a broad catabolic program to restore energy homeostasis, including increased glucose uptake, glycolysis, lipolysis, fatty acid oxidation, and mitochondrial biogenesis, and suppression of hepatic glucose production [18]. Indeed, activation of AMPK in response to exercise or T2D therapeutics such as metformin improves glucose metabolism [19], [20]. We therefore sought to identify amino acids which could both modulate activity of AMPK in a cellular system and improve *in vivo* metabolism. We report that l-alanine (Ala) uniquely increases phosphorylation of AMPK and in parallel improves glucose tolerance *in vivo* in mice.

## 2 Material and methods

### 2.1 Cell culture

H4IIE, Hepa1c, and HepG2 cells were grown in Dulbecco's modified Eagle's medium (DMEM) with 1 g/L d-glucose, 1 mM sodium pyruvate, 10% fetal bovine serum (FBS), and antibiotics (100 UI/L penicillin, 100 μg/mL streptomycin) at 37 °C and 5% CO_2_. Cells at ≈80% confluence were serum starved overnight before treatment with amino acids or other compounds in Dulbecco's phosphate-buffered saline (DPBS) with 4.5 g/L (25 mM) glucose, 6 mM HEPES, 3.7 mM sodium bicarbonate (modified DPBS: MDPBS, Table S1).

### 2.2 Western blot

Cells were lysed in 10 mM Tris-HCl, 4% SDS and 20% glycerol, pH 6.8 with protease and phosphatase inhibitors (Sigma, USA). Liver samples were homogenized in ice-cold RIPA buffer (25 mM Tris-HCl, 150 mM NaCl, 0.1% SDS, 0.5% sodium deoxycholate, 1% NP-40, pH 7.6) with protease and phosphatase inhibitors. Homogenates were centrifuged at 15,000 × *g* for 10 min. Supernatants were stored at −80 °C. Protein concentration was determined by Bradford assay (Bio-Rad, CA, USA). Samples were heat-denatured in sample buffer at 95 °C for 3 min; 20 μg of protein were separated by 7.5% SDS-PAGE and transferred to PVDF for blotting using the following antibodies (Cell Signaling, MA, all 1:1000): anti-p-AMPKα (Thr^172^), anti-p-ACC (Ser^79^), anti-p-mTOR (Ser^2448^), anti-p-70S6K (Thr^389^), anti-p-S6 (Ser^235/236^), anti-AMPKα, anti-ACC, anti-mTOR, anti-p70S6K and anti-S6. Blots were visualized with HRP-conjugated goat anti-rabbit IgG and ECL and quantified using ImageJ. For details of in-cell Westerns, see legend for Figure S1.

### 2.3 Immunohistochemistry

Cells were fixed in 4% formaldehyde for 10 min, rinsed with PBS, incubated in 5% goat serum and then with primary antibodies (1:400, anti-pAMPK rabbit IgG in 5% goat serum) overnight at 4 °C and subsequently with Alexa Fluor 568-conjugated secondary antibody (1:200). Nuclei were stained with DAPI and visualized by confocal microscopy (Carl Zeiss, LSM-710, Germany).

### 2.4 Glucose release and uptake

Glucose release was assessed as previously reported [21]. Briefly, Ala or insulin were added to DPBS with 6 mM HEPES and 3.7 mM sodium bicarbonate and incubated for 3 h. Medium glucose was determined by fluorometric enzymatic assay and normalized to cell number.

Glucose uptake was assayed after overnight serum starvation; medium was replaced with DPBS with HEPES and bicarbonate (pH 7.2) prior to incubation with Ala or insulin for 15 min 2-deoxyglucose (5 mM) and ^3^H-2-deoxyglucose (0.5 μCi/mL) were added for 5 min before washing and lysis. Glucose uptake was determined by scintillation counting, and normalized to protein.

### 2.5 Intracellular NAD^+^/NADH and adenine nucleotides

Cells were incubated with 5 mM Ala or 100 μM rotenone (positive control) for 15 or 30 min. NAD/NADH levels were measured using a commercial kit (BioVision Inc., USA). Adenine nucleotide ratios were determined as previously reported [22] using HPLC with an ACQUITY-UPLC^®^ T3 column (Nihon Waters, Tokyo, Japan) and detection at 290 nm.

### 2.6 Quantitative metabolomics

After stimulation, cells were rinsed with dH_2_0, and immediately frozen in liquid nitrogen prior to lysis with methanol:chloroform:water (1:9:30). Targeted, multiple reaction monitoring MS data acquisition was used to measure water-soluble metabolites in distinct classes. Using the ABI Sciex 5500, coefficients of variation (CV) for analyte measurements are ≤15%. For full description see [23].

### 2.7 Ammonia production

H4IIE cells were incubated with Ala or/and sodium pyruvate for 1 h in MDPBS. Ammonia in conditioned media was measured using Bioprofile FLEX^®^ analyzer (Nova Biomedical (USA) and normalized by cell number.

### 2.8 siRNA-mediated knockdown

ON-TARGETplus smart pool siRNAs for ALT1, LKB1, or non-targeting scrambled control siRNA (all 25 nM) were transfected using 50 nM Dharmafect^®^ (Thermo Scientific, USA). Seventy hours later, cell lysates were prepared for Western blotting.

### 2.9 *In vivo* analyses

C57BL/6J mice were fasted for 4 h prior to oral administration of alanine (1.5 g/kg) dissolved in saline. After 30 min, mice were anesthetized with chloral hydrate (500 mg/kg intraperitoneally), and tissues dissected, immediately frozen in liquid nitrogen, and stored at −80 °C. Oral or intraperitoneal glucose tolerance were performed in C57BL/6J mice fasted for 15 h prior to oral gavage with Ala (1.5 g/kg) and administration of glucose (1 g/kg) 15 min later. Glucose was measured in tail vein blood samples. For high-fat feeding, a 60% high-fat diet was used (Research Diets 12,492). Glucose tolerance was similarly assessed in mice expressing a dominant negative AMP kinase (FVB/N-Tg (Ckm-Prkaa2*D157A)1Ljg/Mmjax, JAX).

### 2.10 Statistical analysis

Data are expressed as mean ± SEM. Analysis was performed by one-way analysis of variance (ANOVA) with Dunnett's multiple comparison post hoc test or student's t-tests using GraphPad Prism for Windows (v5). Statistical significance was set at p < 0.05.

## 3 Results

### 3.1 Ala activates AMP in cultured cells

**Figure 1:**
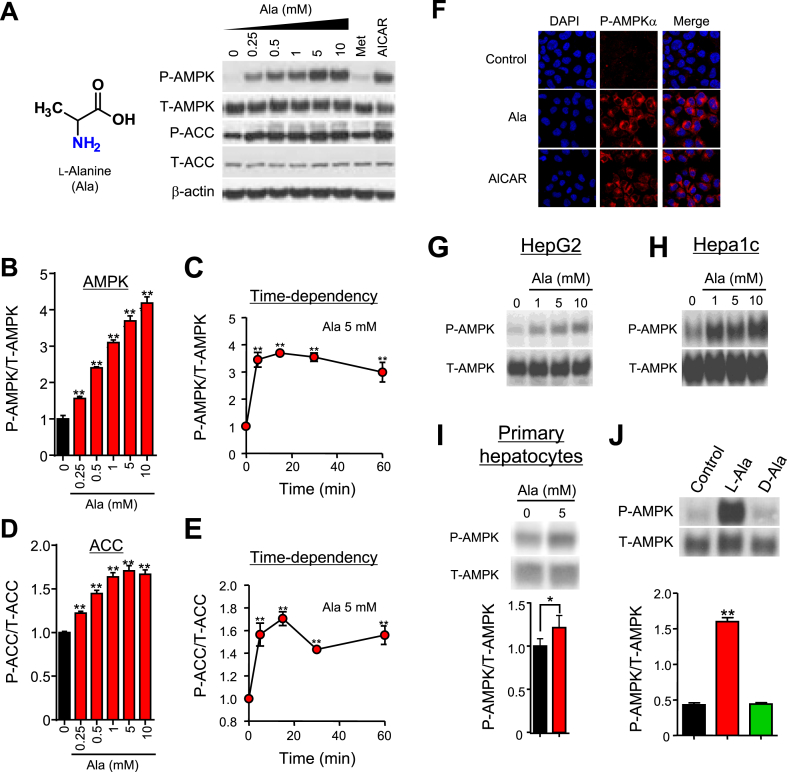
Ala activates AMPK. (A) Representative anti-phospho-AMPK (pAMPK) and phospho-ACC (pACC) Western blots in H4IIE cells treated with Ala, metformin (Met, 2 mM) or AICAR (2 mM) for 15 min. (B–E) Concentration- and time-dependent effects of Ala in H4IIE cells. Data are normalized to total protein (n = 4). (F) Anti-P-AMPK in H4IIE cells treated with Ala or AICAR for 15 min. (G–I) Anti-P-AMPK western blots in Ala-treated HepG2, Hepa1c, or mouse primary hepatocytes. (J) Impact of l-Ala and D-Ala (5 mM, n = 4). All data are indicated as mean ± SEM. *p < 0.05 or **p < 0.01 versus baseline.

To assess the impact of amino acids on AMPK, we incubated confluent cells with a panel of 20 individual amino acids at 1, 5, and 10 mM for 5–120 min (Figure S1A) and analyzed AMPK activation using an in-cell western assay. We observed a striking dose- and time-dependent effect of Ala to increase AMPK phosphorylation in H4IIE hepatic cells. Cysteine induced similar, but less robust, activation; no significant stimulation was observed with other amino acids. Ala-stimulated phosphorylation of AMPK and its downstream effector ACC was confirmed by standard Western blot (Figure 1A) and was similar to effects of the AMPK activators AICAR (5-aminoimidazole-4-carboxamide riboside) and metformin [24]. Ala-induced increases in AMPK phosphorylation were both dose-dependent over a range of 250 μM to 10 mM (1.6–4.2-fold induction, p < 0.01 for all, Figure 1A, B) and time-dependent, peaking at 15 min (4.2-fold, p < 0.001, Figure 1C), and were confirmed using immunofluorescence (Figure 1F). Dose and time-dependent effects on ACC phosphorylation were similarly robust (p < 0.01, Figure 1D, E). Ala also increased AMPK phosphorylation in human HepG2, mouse Hepa1c, and primary mouse hepatocytes (Figure 1G–I), with similar dose response and time course (not shown). D-Ala did not activate AMPK (Figure 1J). Ala similarly activated AMPK in serum-replete or starved conditions (Figure S1B, C) and in both low (5.5 mM) and high (25 mM) glucose (Figure S1D). Thus, Ala activates AMPK under a variety of nutrient conditions.

### 3.2 Ala increases AMPK phosphorylation and reduces glucose *in vivo*

**Figure 2:**
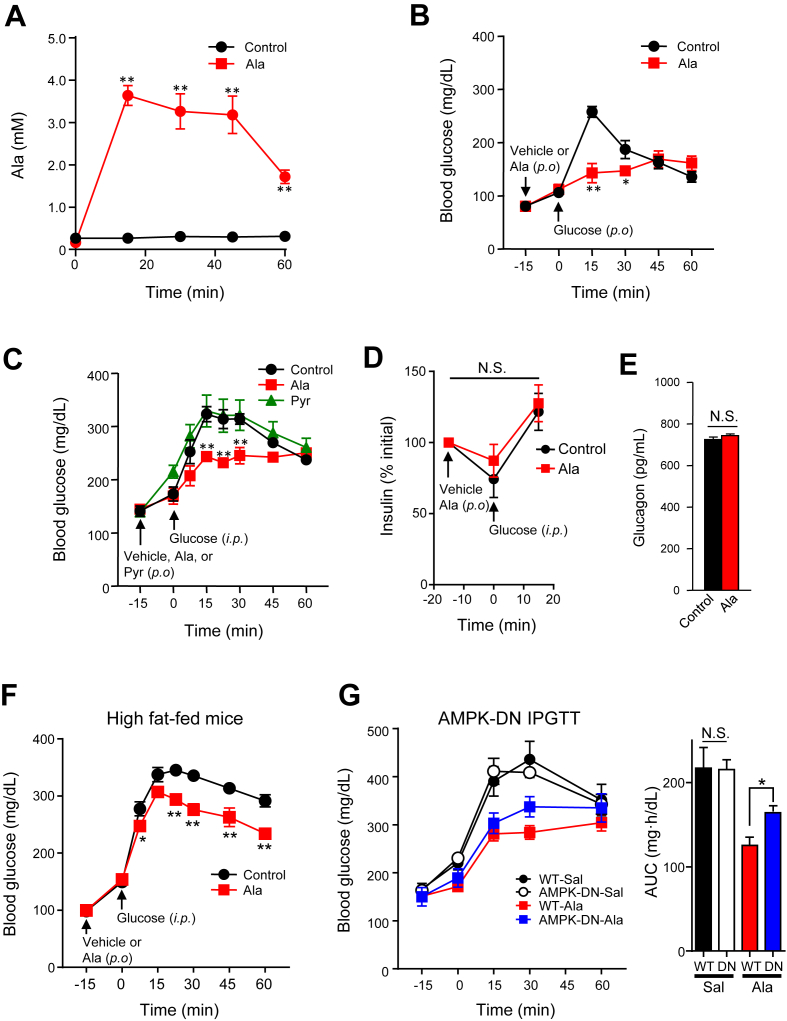
Ala gavage increases plasma Ala and improves glucose tolerance in both chow and HFD-fed mice. (A) Plasma Ala after oral administration (1.5 g/kg, n = 5). (B) Oral glucose tolerance in control or Ala-gavaged mice (1 g/kg 15 min before glucose, n = 6/group). (C) Intraperitoneal glucose tolerance in control, Ala- or Pyr-gavaged mice (1.5 g/kg 15 min prior to glucose, n = 6/group). (D) Plasma insulin and (E) glucagon in control and Ala-treated mice during IPGTT (n = 6/group). (F) Blood glucose in high fat-fed mice gavaged with Ala (1.5 g/kg 15 min before IPGTT, n = 8/group). (G) IPGTT in AMPK-DN or wild type mice after oral Ala (1.5 g/kg 15 min before IP glucose, n = 8/group). All data are indicated as mean ± SEM. *p < 0.05 or **p < 0.01 versus control mice.

We next asked whether Ala could also activate AMPK *in vivo*. Oral gavage with Ala (1.5 g/kg) resulted in sustained increases in plasma levels over 60 min, with a peak of 3.6 mM at 15 min (Figure 2A). In parallel, Ala gavage increased phosphorylation of AMPK by 1.5 fold in liver at 30 min (p < 0.05 vs. control, Figure S2A), with similar trends for increased ACC phosphorylation. There was a trend for increased AMPK (p = 0.08 vs. control, Figure S2B) and significantly increased ACC phosphorylation in epididymal adipose tissue (p < 0.05). AMPK and ACC phosphorylation did not differ at 30 min after Ala administration in quadriceps muscle (Figure S2C).

Since AMPK activation likely contributes to glucose lowering by metformin and other pharmacologic agents, we assessed impact of Ala on blood glucose *in vivo*. Ala gavage prior to oral glucose significantly reduced blood glucose at both 15 and 30 min (OGTT, Figure 2B), patterns similar to effects of metformin (Figure S3A). Ala also significantly reduced glucose at 15, 30, and 45 min after intraperitoneal glucose (IPGTT, Figure 2C); by contrast, pre-treatment with pyruvate had no effect on glucose tolerance. Ala-induced reductions in glucose were not accompanied by differences in plasma insulin (Figure 2D), glucagon (Figure 2E), or FGF21 (not shown). Strikingly, Ala gavage also significantly improved glucose tolerance in mice made insulin resistant by high-fat feeding (Figures 2F and S3B, C). Interestingly, responsiveness to Ala was modestly reduced in mice expressing dominant negative AMPK (AMPK-DN) predominantly in skeletal muscle (Figure 2G, right) despite no differences in either GLP1 or insulin (Figure S3D, E).

### 3.3 Ala suppresses the mTOR pathway and affects glucose metabolism

**Figure 3:**
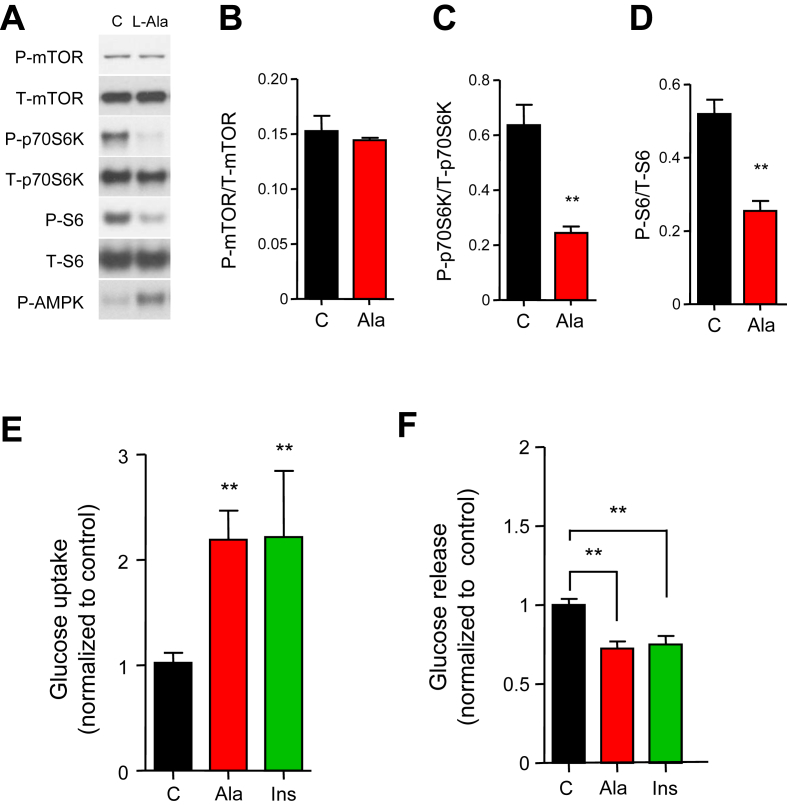
Effects of Ala on mTOR pathway and glucose metabolism in H4IIE cells. (A) Representative western blots in cells treated with 5 mM Ala for 15 min (B–D) Quantification of mTOR, p70S6K and S6 phosphorylation (n = 4). (E) Effects of Ala on ^3^H-deoxyglucose uptake, measured 15 min after Ala incubation (n = 6). (F) Effects of Ala on glucose output, measured in conditioned medium after incubation with 5 mM Ala or 100 nM insulin (Ins) for 4 h (n = 6/group). All data are presented as mean ± SEM. **p < 0.01 versus control.

Consistent with AMPK inhibition of mTOR [18], Ala reduced downstream Tor pathway effectors p70S6 kinase and ribosomal protein S6 phosphorylation by 38% and 49%, respectively in H4IIE cells (p < 0.01), both at baseline (Figure 3A–D) and after treatment with insulin or leucine (Figure S4A, B).

Ala activation of AMPK and suppression of mTOR signaling in the liver would be expected to increase glucose uptake [24], [25] and suppress glucose output [26], [27], [28]. Indeed, acute Ala significantly increased glucose uptake (2.2 fold, p < 0.05, Figure 3E) and suppressed glucose release by 65% (p < 0.01), a magnitude similar to the effect of insulin (57% suppression, p < 0.01, Figure 3F).

### 3.4 Effects of Ala on upstream activators of AMPK

We next examined potential upstream mechanisms by which Ala activates AMPK. AMPK can be activated by osmotic stress [29], [30], [31], [32]; for example, the hypertonic compound mannitol increases AMPK phosphorylation in parallel with reduced cell size and increased intracellular calcium (Figure S4C, D). However, Ala did not alter cell size or intracellular calcium (Figure S4C, D), and equi-osmolar concentrations of d-alanine did not activate AMPK (Figure 1J).

We next examined whether Ala regulates LKB1, a major upstream AMPK activator regulated by phosphorylation [18] and cytosolic translocation [33]. Ala minimally increased LKB1 phosphorylation (12% at 5 mM, p < 0.05, Figure S4E), but did not alter its translocation (Figure S4F). siRNA knockdown of LKB1 reduced basal AMPK, but did not alter Ala-stimulated AMPK phosphorylation (Figure S4G–I). Thus, it is unlikely that LKB1 contributes to Ala-mediated AMPK activation.

Conversely, we examined whether Ala could inhibit kinases known to inhibit AMPK activity, such as Akt and PKA [34], [35]. As previously shown, Ala reduced activation of mTOR targets, both at baseline (Figure 3) and after insulin or leucine (Figure. S4A, B). However, neither insulin nor leucine modified Ala-induced AMPK phosphorylation. Similarly, Ala effects were not altered by rapamycin (inhibitor of mTOR signaling) or Br-cAMP or glucagon (activators of PKA), indicating these pathways were not required (not shown).

### 3.5 Ala alters intracellular metabolites

**Figure 4:**
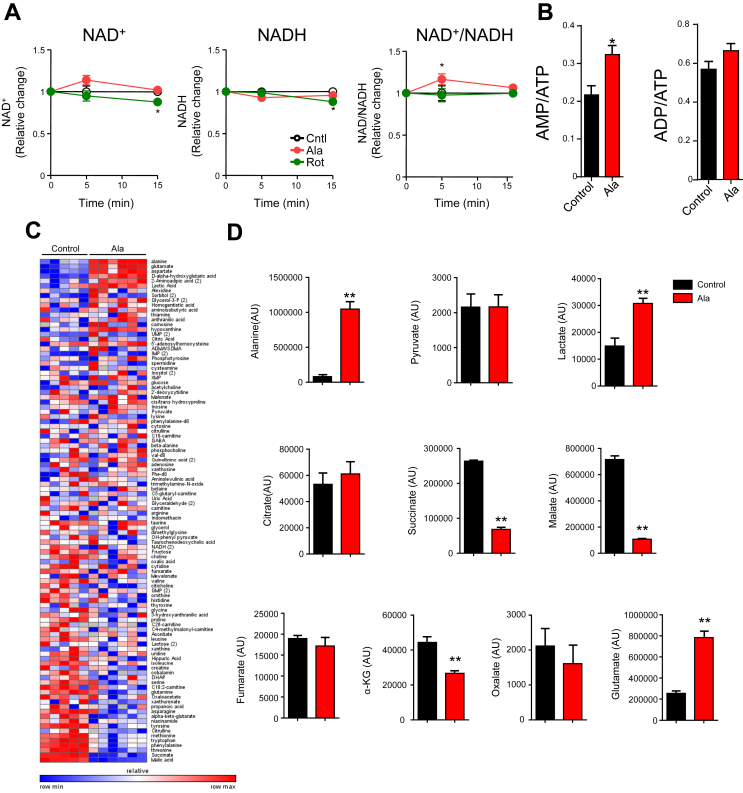
Ala alters cellular metabolism in H4IIE cells. (A) Effects of Ala (5 mM) and rotenone (100 uM, positive control) on intracellular nicotinamides (n = 4). (B) Effects of 5 mM Ala on adenine nucleotides (n = 6). (C) Heat map of metabolomic data from control and Ala-treated cells (Ala 5 mM, 15 min). Red indicates higher levels, while green indicates lower levels relative to median of row. (D) Changes in individual metabolites (n = 5 or 6 per group). All data are expressed as mean ± SEM. *p < 0.05, **p < 0.01 versus baseline.

AMPK is potently activated by increased AMP/ATP, ADP/ATP, or NAD+/NADH ratios [18]. Ala significantly increased NAD+/NADH ratio at 5 min (Figure 4A). These modest effects are unlikely to be responsible for Ala-mediated AMPK activation, as they were not sustained (while AMPK activation was observed for up to 2 h). By contrast, Ala robustly increased the AMP/ATP ratio by 1.5-fold at 15 min (p < 0.05, Figure 4B).

To identify Ala-mediated changes in metabolism potentially contributing to increased AMP/ATP ratio, we analyzed H4IIE cell extracts using a LC-MS/MS-based metabolomics platform. Of 104 metabolites measured (Figure 4C), 25 were regulated (7 increased, 18 decreased, nominal p < 0.05). Ala was increased by 13-fold (p < 0.0001, Figure 4C, D). Although Pyr levels did not differ, lactate levels were significantly increased. Interestingly, Ala markedly reduced the TCA cycle intermediates succinate, malate, and α-ketoglutarate (αKG) (Figure 4D).

*In vivo* metabolomics (Figure S5A) revealed significantly increased AMP/ATP and ADP/ATP ratios in Ala-treated liver (Figure S5B), indicating altered energetics similar to Ala-treated cells (Figure 4B), with similar trends for decreased αKG and malate. Ala, glutamate, aspartate, glutamine, and asparagine were similarly upregulated in both tissues and cells (Figure S5C).

### 3.6 Supplementation with pyruvate or TCA cycle intermediates abolishes Ala-mediated AMPK activation

**Figure 5:**
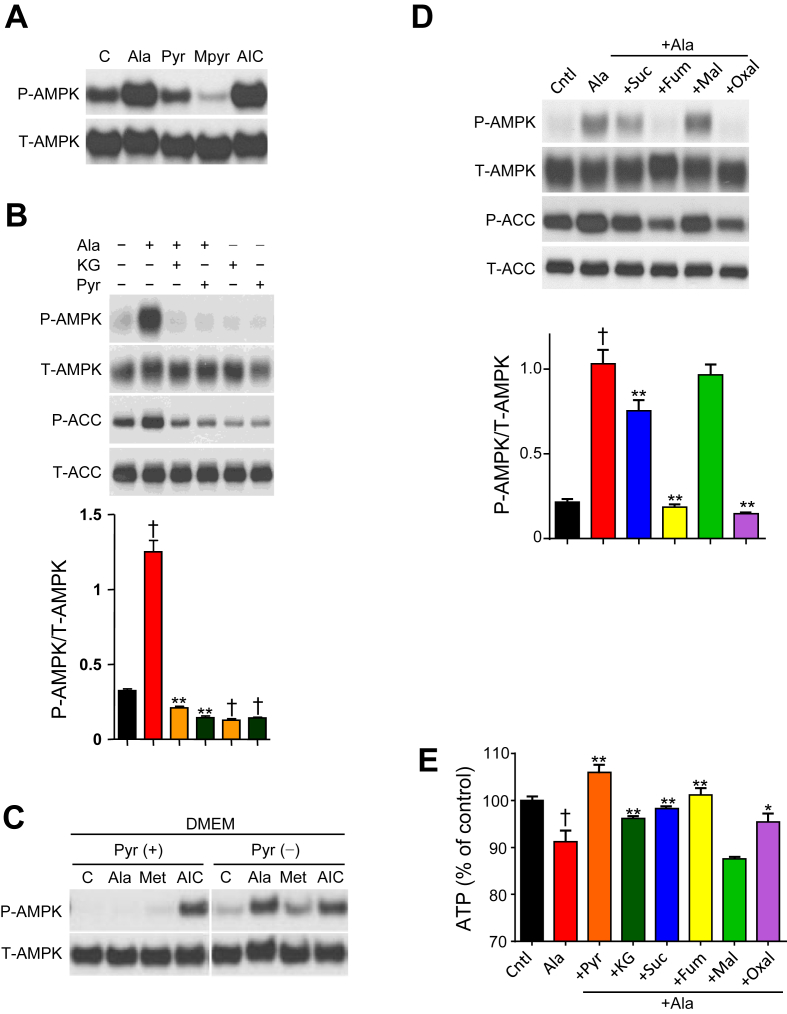
Pyruvate and α-ketoglutarate (α-KG) abolish Ala-mediated AMPK activation in H4IIE cells. (A) Representative anti-p-AMPK western blot in cells treated with Ala, 1 mM Pyr, or 1 mM methylpyruvate. (B) Effect of pyruvate or α-KG (1 mM each, n = 6). (C) Effect of Ala on p-AMPK in DMEM with or without Pyr. (D,E). Anti-p-AMPK blot and intracellular ATP levels after Ala treatment (15 min) with methyl succinate (Suc, 1 mM), methylfumarate (Fum, 1 mM), methylmaleate (Mal, 1 mM), oxaloacetate (Oxal, 10 mM) (n = 5–6). All data are expressed as mean ± SEM. *p < 0.05, **p < 0.01 versus Ala, ^†^p < 0.01 versus control.

Since Ala-induced reduction in TCA cycle intermediates could contribute to reduced ATP and activation of AMPK, we tested whether supplementation with pyruvate or TCA cycle intermediates could reverse the impact of Ala. Neither Pyr nor cell-permeable methylpyruvate activated AMPK (Figure 5A), but supplementation with Pyr abolished Ala-mediated AMPK activation (Figure 5B). Interestingly, inclusion of Pyr in culture medium inhibited both Ala and metformin-induced activation of AMPK; AICAR was still able to activate AMPK phosphorylation (Figure 5C). TCA cycle intermediates, including αKG, succinate, fumarate, and oxaloacetate, also reduced Ala effects (Figure 5D). In parallel, both Pyr and TCA cycle intermediates restored intracellular ATP (Figure 5E).

### 3.7 Ala and ammonia metabolism play significant roles in AMPK activity

Ala is primarily metabolized to Pyr via alanine transaminase 1 (ALT1) in liver. Since neither Pyr nor methyl-Pyr (cell permeable) activated AMPK in H4IIE cells (Figure 5A), we hypothesized that ALT-mediated Ala metabolism was required for AMPK activation. To test this possibility, we treated cells with ALT1 or scrambled control siRNA; ALT1 siRNA reduced ALT protein by 77% (p < 0.001, Figure S6A). Ala-stimulated AMPK activation was reduced by approximately 49% in ALT1 siRNA-treated cells, despite efficacy of AICAR to stimulate AMPK activation (Figure S6B). Thus, Ala activation of AMPK requires ALT-mediated metabolism.

**Figure 6:**
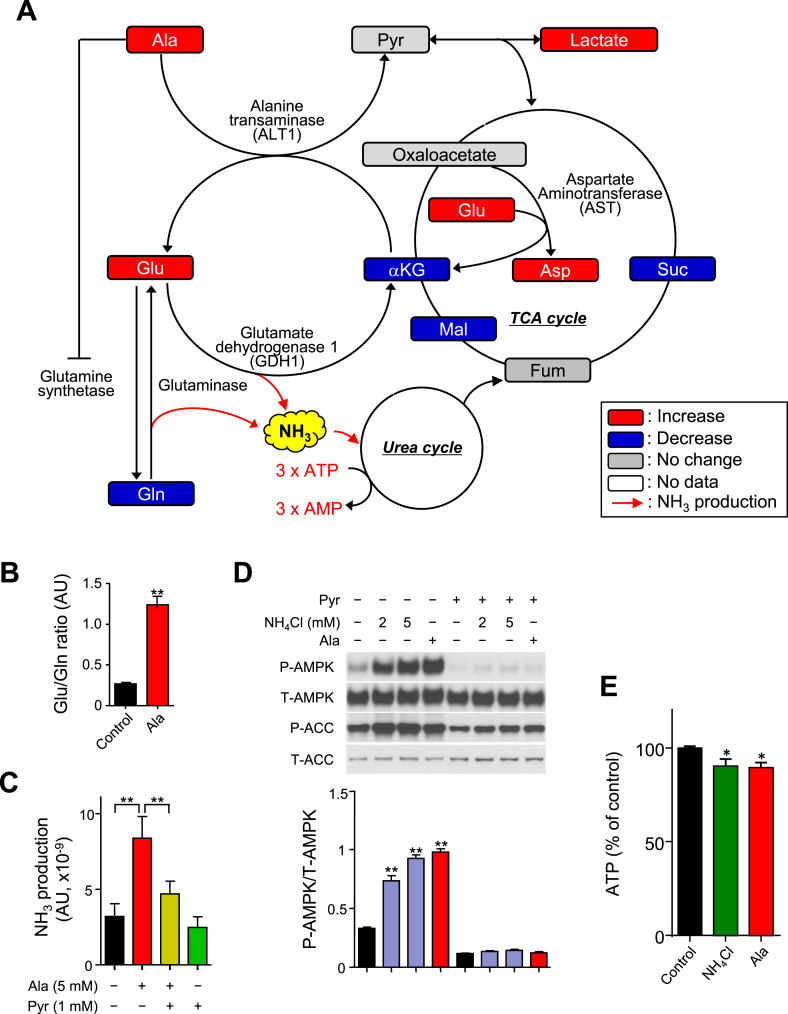
Ammonia metabolism contributes to regulation of AMPK. (A) Ala metabolic pathway overlaid with metabolite data in H4IIE cells treated with 5 mM Ala for 15 min. Red and blue indicate metabolites increased and decreased by Ala, respectively. (B) Glu/Gln ratio in H4IIE cells treated with Ala (n = 5–6 per group). (C) Ammonia production in H4IIE cells treated with Ala ± Pyr for 1 h (n = 8). (D) Representative western blot and quantitation of p-AMPK in H4IIE cells treated with NH_4_Cl ± 1 mM Pyr. (E) Intracellular ATP levels in H4IIE cells treated with NH_4_Cl for 15 min (n = 4). All data are expressed as mean ± SEM. *p < 0.05, **p < 0.01.

ALT catalyzes formation of glutamate from αKG as Ala is metabolized to pyruvate. Consistent with this mechanism, Ala significantly increased glutamate and reduced αKG (Figures 4D and 6A). Thus, Ala metabolism may consume αKG to form glutamate, reducing TCA cycle intermediates and ATP production. Given that ALT1 knockdown does not fully inhibit Ala activation, particularly at the 5 mM concentration (Figure S6), we considered additional mechanisms contributing to the effects of Ala. Ala potently increased the glutamate/glutamine ratio (Figure 6B), a likely result of both ALT1-mediated generation of glutamate and indirect effects of allosteric inhibition of glutamine synthase by Ala [36], [37].

Increased glutamate levels could promote AST-mediated generation of aspartate; indeed, aspartate was increased by 12 fold in Ala-treated cells (third-ranked metabolite, Figure 4C). Additionally, deamination of glutamate by glutamate dehydrogenase (GDH) yields NH_3_ [38], [39]; NH_3_ production was increased by 2.6 fold in Ala-treated cells (p < 0.001). Moreover, incubation of cells with NH_4_Cl (2–5 mM) increased phosphorylation of both AMPK and ACC (Figure 6D) and reduced ATP levels (Figure 6E). These effects were inhibited by pyruvate (Figure 6C, D).

## 4 Discussion

Using a high-throughput cell screening approach, we find that Ala is unique among amino acids in its ability to acutely and potently activate AMPK. This is interesting as alanine is recognized as the principal amino acid released from muscle, which regulates inter-organ glucose homeostasis via the glucose-alanine cycle [40]. During short-term starvation or acute exercise, muscle generates Ala via transamination of pyruvate or protein degradation, increasing plasma Ala [40], [41]. In the liver, Ala can also be used as a substrate for gluconeogenesis.

Our findings now suggest a novel role for Ala as a nutrient sensor. *In vivo* Ala administration robustly activates AMPK in the liver, and in parallel, reduces blood glucose in mice during early phases of either oral or IP glucose tolerance testing. These effects appear to result from acute Ala metabolism (Graphical Abstract).

Hepatic AMPK is activated in response to energetic stress (e.g. exercise, fasting) [42], adiponectin [43], and antidiabetic drugs such as metformin [44] and SGLT2 inhibitors [20]. AMPK activation initiates a transcriptional and signaling program which promotes increased energy availability, via both increases in catabolic pathways (e.g. glucose and lipid oxidation [45]) and inhibition of energy-requiring anabolic pathways (e.g. glycogen, lipid, and protein synthesis) [18], [42]. Both pharmacologic and genetic activation of AMPK suppresses hepatic glucose production [28], [46]; conversely, AMPK inhibition via AMPKα2 subunit deletion in liver increases glucose production and ablates adiponectin-mediated signaling [47]. Hepatic AMPK activation also inactivates glycogen synthase, acutely favoring glycolysis and reducing gluconeogenesis [48].

We now demonstrate that Ala activates AMPK and AMPK-dependent downstream pathways, increasing phosphorylation of ACC and inhibiting phosphorylation of mTOR targets p70 S6 kinase and ribosomal protein S6. Consistent with the effects of AMPK activation, Ala acutely suppresses glucose release and increases glucose uptake in cultured hepatoma cells.

Our data indicate that Ala activation of AMPK is mediated via cellular metabolism. The first step of Ala metabolism is ALT1-catalyzed transamination, transferring the amino group to αKG to yield glutamate. Metabolomics analysis demonstrated marked elevations in glutamate and reductions in αKG in Ala-treated cells. Moreover, transamination was also required for Ala-induced AMPK activation; cells treated with an ALT1 siRNA had reduced Ala-induced AMPK activation. We also observed a marked increase in aspartate, potentially reflecting AST-mediated transfer of amino groups from glutamate to oxaloacetate, generating aspartate as a nitrogen source for urea synthesis. While metabolomics data cannot predict flux, these data suggest that Ala metabolism may both deplete TCA cycle intermediate pools and promote ATP-requiring urea synthesis, both of which may contribute to increased AMP/ATP ratio. Consistent with this hypothesis, supplementation with either pyruvate or TCA cycle intermediates not only increased ATP levels, but also reversed Ala-induced AMPK phosphorylation.

Glutamate metabolism and NH_3_ generation appear to play central roles in Ala AMPK activation. Ala increased glutamate concentrations, likely a result of ALT-mediated transamination, and increased glutamate/glutamine ratios. Further metabolism of glutamate via transamination likely contributed to Ala-induced increases in aspartate, both *in vivo* and *in vitro*. Glutamate also undergoes rapid oxidative deamination (by glutamate dehydrogenase), potentially contributing to the robust increase in NH_3_ production after Ala supplementation.

Increased NH_3_ may contribute to indirect effects of Ala; incubation with NH_4_Cl recapitulated Ala-induced increases in the AMP/ATP ratio and increased AMPK and ACC phosphorylation. Like Ala, effects of NH_4_Cl were inhibited by pyruvate. Potential mediators include NH_3_-mediated increases in urea synthesis (an ATP-requiring process) or induction of autophagy, as shown in glucose-deprived cells [49]; these effects are similar to Ala effects, occurring via an mTOR-independent mechanism, and are inhibited by pyruvate.

Together, our data suggest that acute metabolism of Ala, including transamination and oxidative deamination, results in depletion of TCA cycle intermediates and generation of NH_3_, both of which alter cellular energetics and promote AMPK activation. These pathways may be particularly critical during energetic stress, such as fasting and/or exercise, when glucose and pyruvate availability are limited. Further studies will be required to assess cellular flux through these pathways in response to acute Ala.

Metabolic effects of Ala are also observed *in vivo*. Oral Ala administration not only activates AMPK, but also reduces plasma glucose during both oral and intraperitoneal glucose tolerance testing. Strikingly, Ala also improved glucose tolerance in both chow-fed controls and HFD-fed insulin resistant mice. Our data indicate that Ala activation of AMPK was most robust in the liver, and thus is the most likely tissue contributing to these effects. However, we did observe modest reduction in the glucose-lowering effects of acute Ala in mice with muscle-specific inactivation of AMPK, suggesting muscle AMPK also contributes to Ala-induced improvement in systemic glucose tolerance. It is possible that our analysis of AMPK phosphorylation at a single time point in quadriceps muscle was not sufficient to detect earlier and perhaps transient activation in this tissue. While our studies in hepatic cells suggest that effects of Ala are likely to be largely cell autonomous, further studies will be required to identify additional physiologic responses in not only liver, but also muscle and other tissues, which may contribute to Ala-induced activation of AMPK and improvements in glucose tolerance.

Taken together, our data suggest a new role for Ala as an energetic sensor which activates the AMPK pathway and acutely reduces blood glucose. These effects are likely to be particularly relevant during conditions of energetic stress, such as fasting or exercise, when circulating Ala levels are increased. Ala and downstream metabolites modulate cellular energetics, thus promoting AMPK-dependent catabolic responses to restore energy homeostasis. Sustained elevations in Ala may also enhance gluconeogenesis, as described in the classical glucose-alanine cycle, ultimately promoting restoration of muscle energetics. Future studies will be needed to determine whether impairments in Ala/NH_3_ metabolism and AMPK induction contribute to susceptibility to metabolic disease, and to define whether intermittent administration of Ala could be used therapeutically to enhance glucose tolerance.

## Author contribution

YA, ALD, IH, CA, SW, XS, KNG, NGH, HT, and CD performed the research and analyzed the data. YN, LJG, MCH, REG, and MEP supervised the research and edited the manuscript. YA, ALD, and MEP wrote the manuscript. Funding sources had no role in the collection, analysis or interpretation of data, writing of the manuscript, or submission.
