# Supplementary material for: l-Alanine activates hepatic AMP-activated protein kinase and modulates systemic glucose metabolism
Source: Mol Metab. 2018 Aug 11;17:61–70. doi: 10.1016/j.molmet.2018.08.002 (PMC6197624; doi:10.1016/j.molmet.2018.08.002)
Supplement: Multimedia component 2 [file mmc2.pptx]

## Slide 1
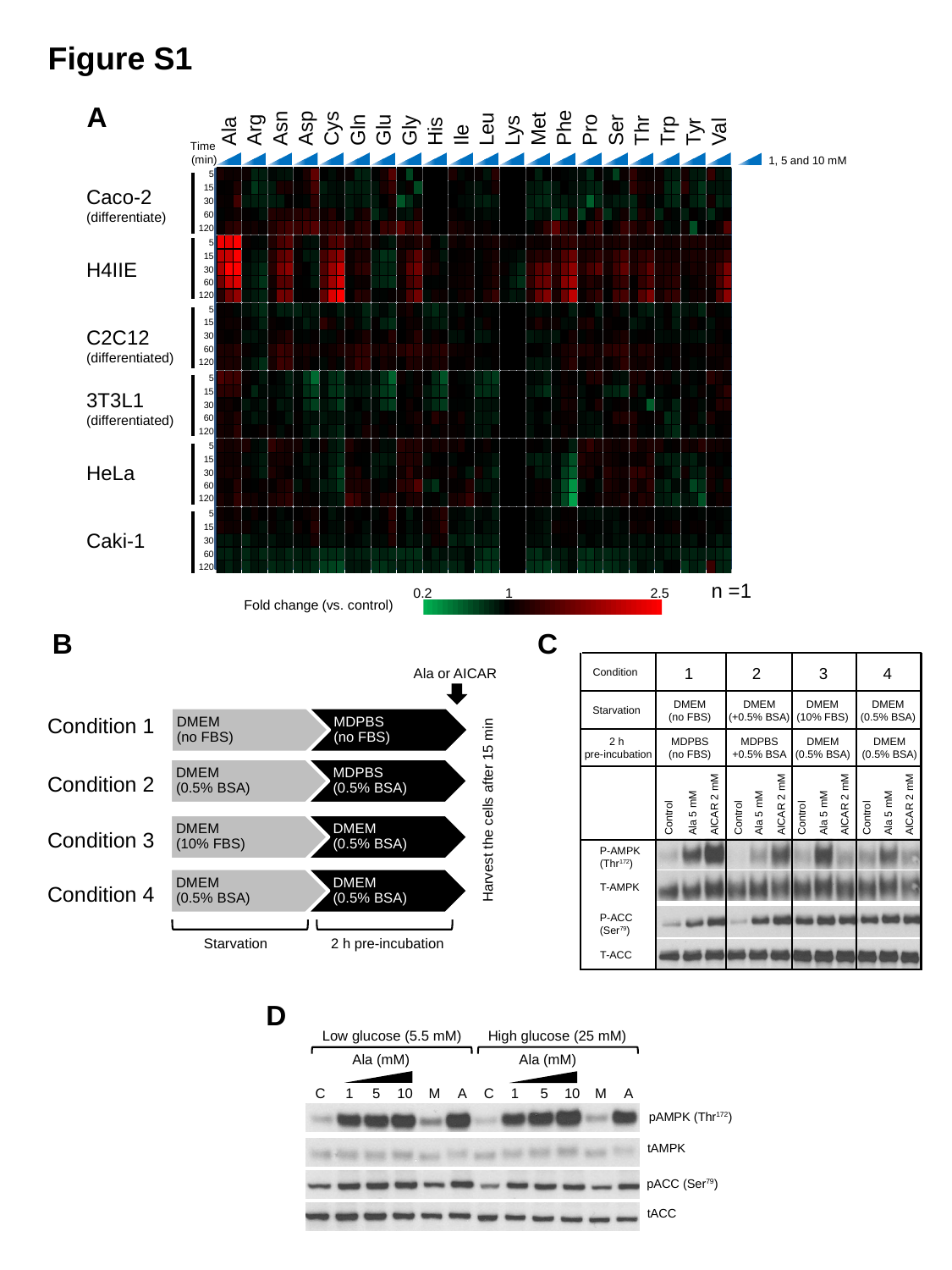

Figure S1
A
C
B
1
2
3
4
Ala or AICAR
Condition
DMEM
(no FBS)
DMEM
(+0.5% BSA)
DMEM
(10% FBS)
DMEM
(0.5% BSA)
Starvation
Condition 1
MDPBS
(no FBS)
MDPBS
+0.5% BSA
DMEM
(0.5% BSA)
DMEM
(0.5% BSA)
2 h
pre-incubation
Condition 2
AICAR 2 mM
AICAR 2 mM
AICAR 2 mM
AICAR 2 mM
Harvest the cells after 15 min
Ala 5 mM
Ala 5 mM
Ala 5 mM
Ala 5 mM
Control
Control
Control
Control
Condition 3
P-AMPK
(Thr172)
T-AMPK
Condition 4
P-ACC
(Ser79)
Starvation
2 h pre-incubation
T-ACC
D
Low glucose (5.5 mM)
High glucose (25 mM)
Ala (mM)
Ala (mM)
C
1
5
10
M
A
C
1
5
10
M
A
pAMPK (Thr172)
tAMPK
pACC (Ser79)
tACC

## Slide 2
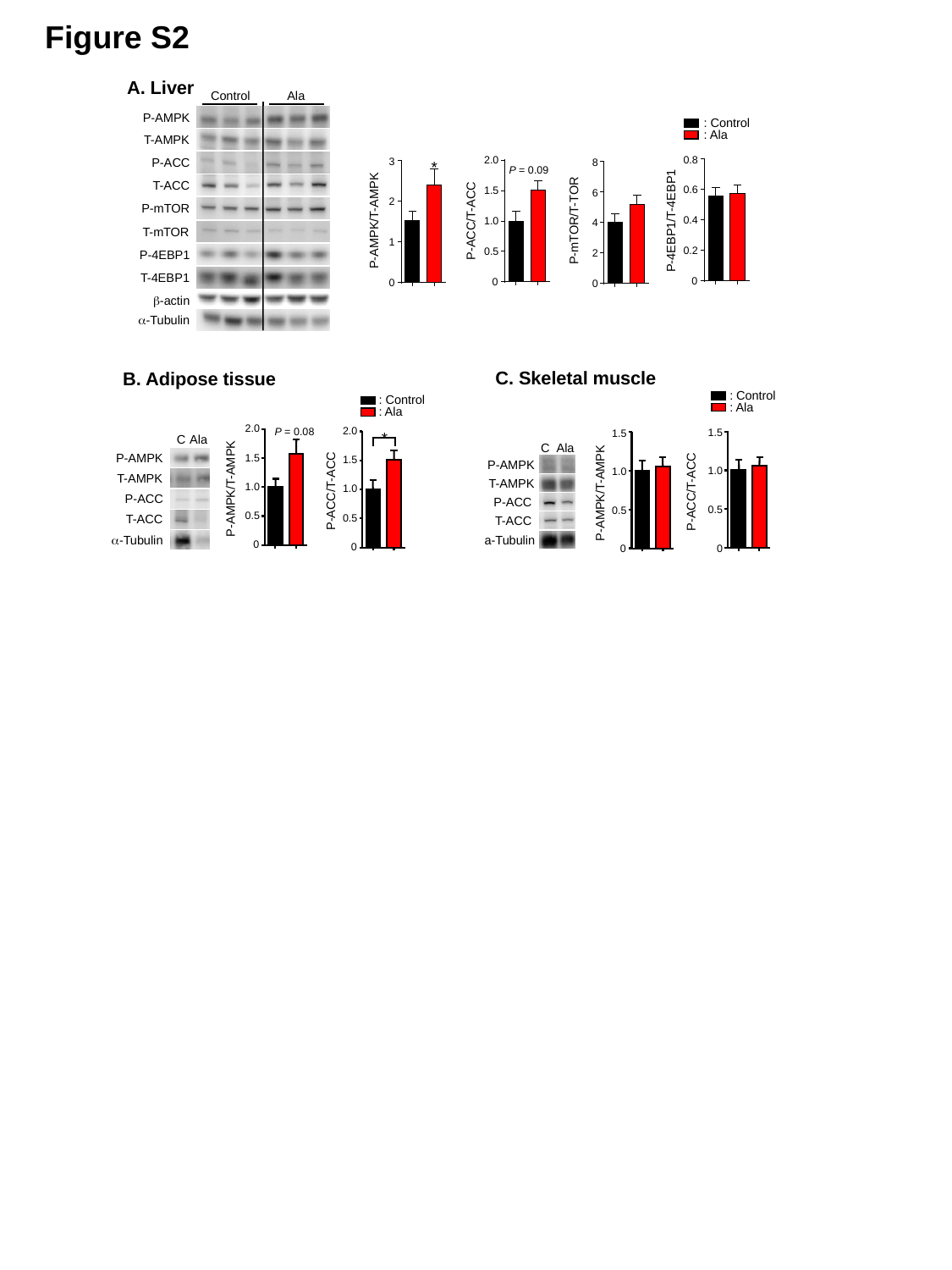

Figure S2
A. Liver
Control
Ala
P-AMPK
: Control
: Ala
T-AMPK
P-ACC
*
0.8
2.0
3
8
P = 0.09
T-ACC
0.6
1.5
6
P-mTOR
2
P-AMPK/T-AMPK
P-ACC/T-ACC
P-mTOR/T-TOR
P-4EBP1/T-4EBP1
0.4
1.0
4
T-mTOR
1
P-4EBP1
0.2
0.5
2
T-4EBP1
0
0
0
0
b-actin
a-Tubulin
C. Skeletal muscle
B. Adipose tissue
: Control
: Control
: Ala
: Ala
P = 0.08
2.0
2.0
C
Ala
1.5
1.0
P-ACC/T-ACC
0.5
0
1.5
*
C
Ala
P-AMPK
P-AMPK
1.5
1.5
T-AMPK
1.0
T-AMPK
P-AMPK/T-AMPK
P-ACC/T-ACC
P-AMPK/T-AMPK
1.0
1.0
P-ACC
P-ACC
0.5
T-ACC
T-ACC
0.5
0.5
a-Tubulin
a-Tubulin
0
0
0

## Slide 3
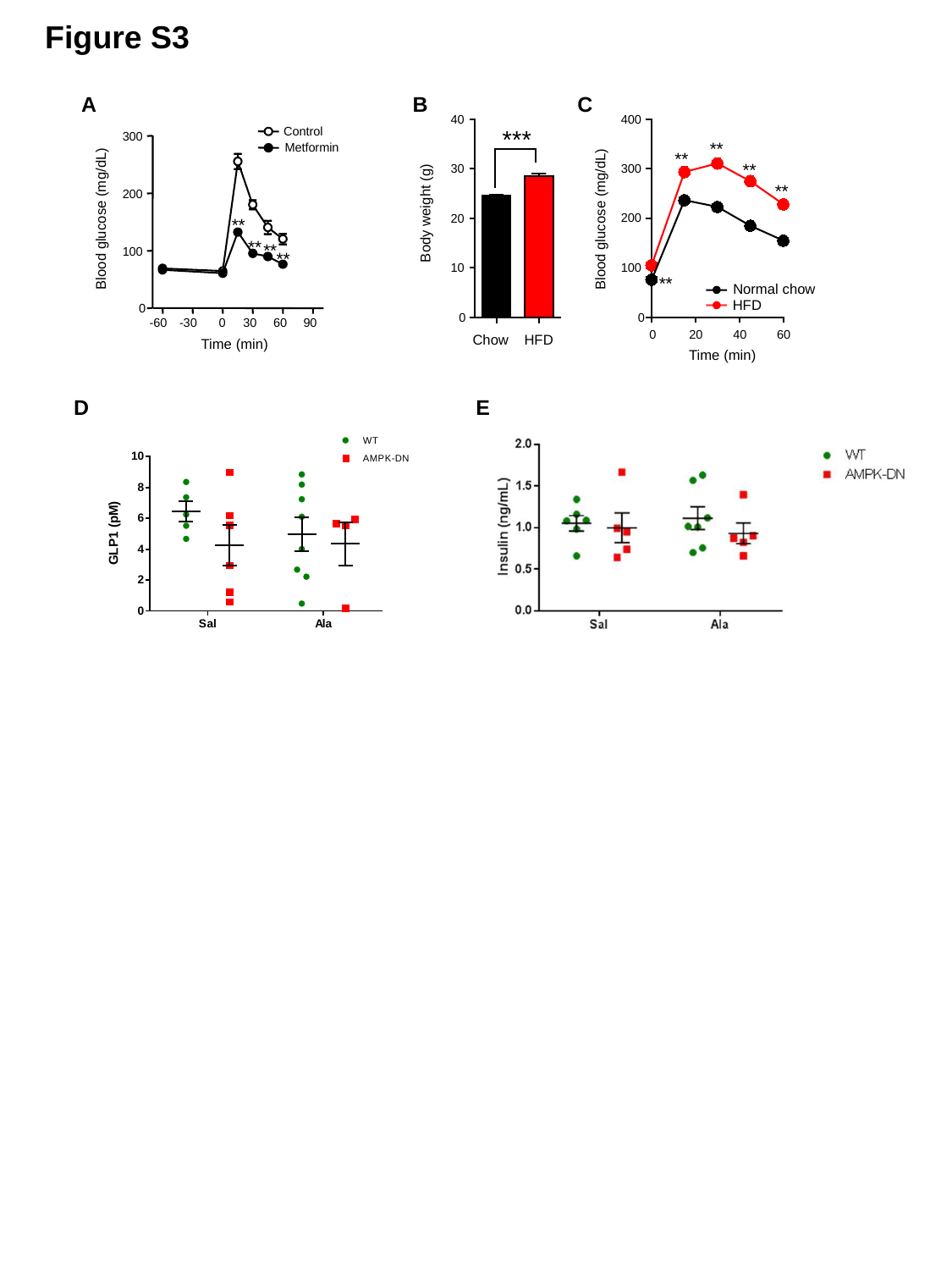

Figure S3
A
B
C
40
400
Control
***
300
**
Metformin
**
**
30
300
**
200
Body weight (g)
Blood glucose (mg/dL)
200
20
Blood glucose (mg/dL)
**
**
**
100
**
100
10
**
Normal chow
HFD
0
0
0
-60
-30
0
30
60
90
Chow
HFD
0
20
40
60
Time (min)
Time (min)
D
E

## Slide 4
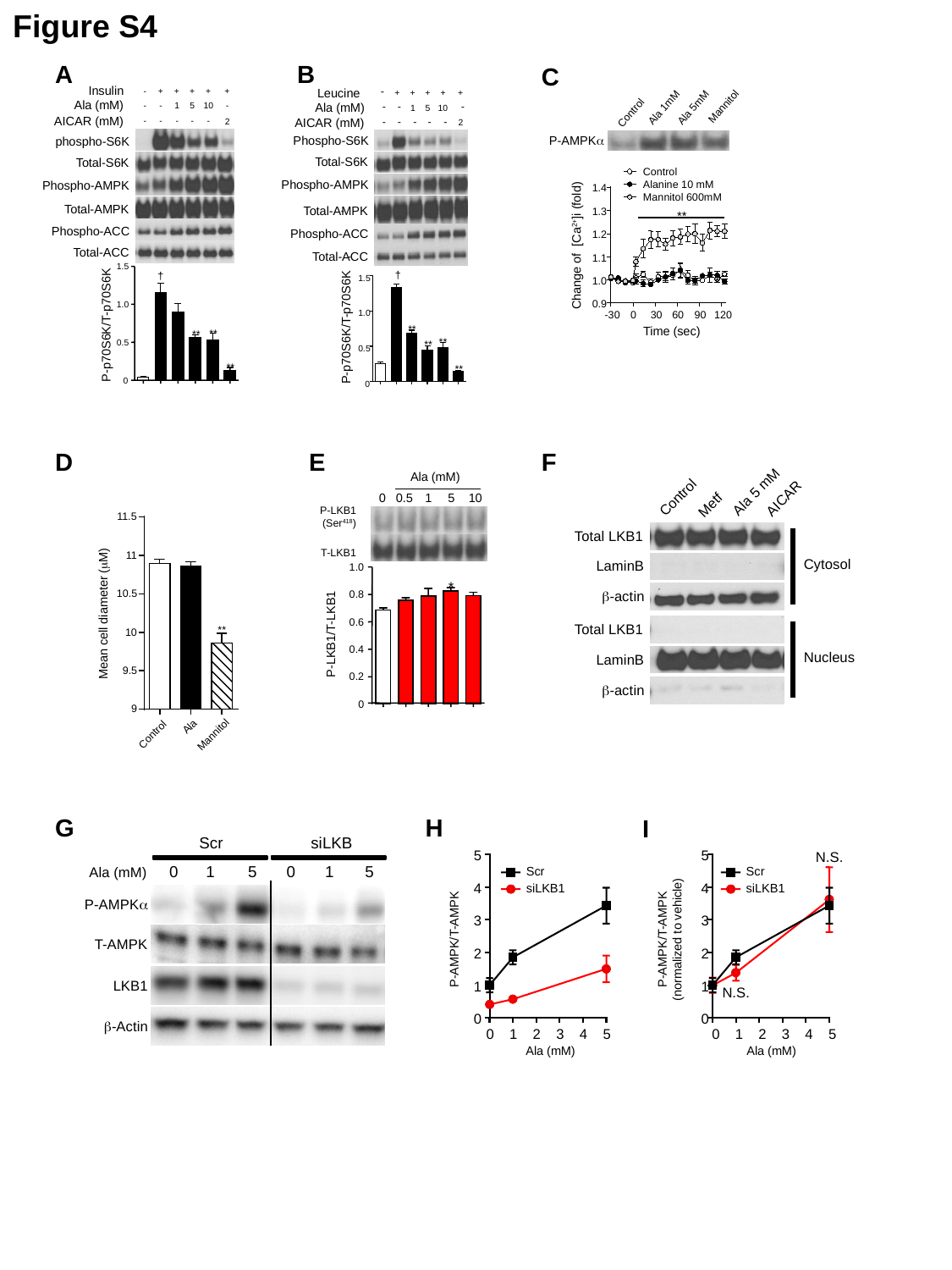

Figure S4
A
B
C
Mannitol
Ala 1mM
Ala 5mM
Control
P-AMPKa
Control
Alanine 10 mM
1.4
Mannitol 600mM
**
1.3
1.2
Change of [Ca2+]i (fold)
1.1
1.0
0.9
-30
0
30
60
90
120
Time (sec)
Insulin
-
+
+
+
+
+
-
Leucine
+
+
+
+
+
Ala (mM)
-
-
1
5
10
-
Ala (mM)
-
-
-
1
5
10
AICAR (mM)
-
-
-
-
-
2
AICAR (mM)
-
-
-
-
-
2
phospho-S6K
Phospho-S6K
Total-S6K
Total-S6K
Phospho-AMPK
Phospho-AMPK
Total-AMPK
Total-AMPK
-
Phospho-ACC
Phospho-ACC
Total-ACC
Total-ACC
-
1.5
†
†
1.5
1.0
1.0
P-p70S6K/T-p70S6K
P-p70S6K/T-p70S6K
**
**
**
**
0.5
**
0.5
**
**
0
0
D
E
F
Ala (mM)
Ala 5 mM
Control
AICAR
0
0.5
1
5
10
Metf
P-LKB1
(Ser418)
11.5
11
10.5
Mean cell diameter (mM)
**
10
9.5
9
Ala
Control
Mannitol
Total LKB1
T-LKB1
Cytosol
LaminB
1.0
*
b-actin
0.8
Total LKB1
0.6
P-LKB1/T-LKB1
Nucleus
0.4
LaminB
0.2
b-actin
0
G
H
I
Scr
siLKB
5
5
N.S.
0
1
5
0
1
5
Ala (mM)
Scr
Scr
4
4
siLKB1
siLKB1
P-AMPKa
3
3
P-AMPK/T-AMPK
(normalized to vehicle)
P-AMPK/T-AMPK
T-AMPK
2
2
LKB1
1
1
N.S.
0
0
b-Actin
0
1
2
3
4
5
0
1
2
3
4
5
Ala (mM)
Ala (mM)

## Slide 5
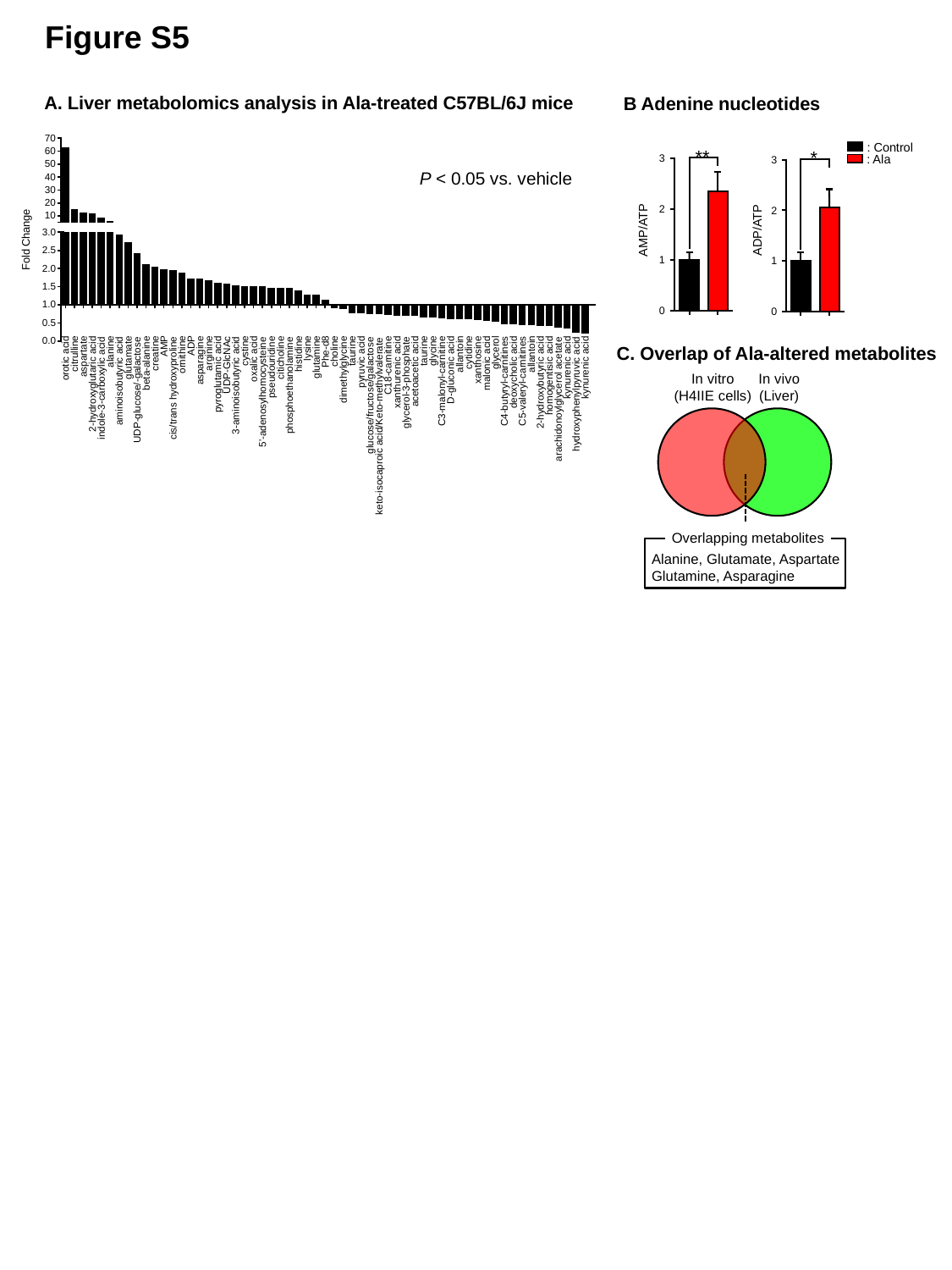

Figure S5
A. Liver metabolomics analysis in Ala-treated C57BL/6J mice
B Adenine nucleotides
: Control
: Ala
**
*
3
3
P < 0.05 vs. vehicle
2
2
AMP/ATP
ADP/ATP
1
1
0
0
C. Overlap of Ala-altered metabolites
In vitro
(H4IIE cells)
In vivo
(Liver)
Overlapping metabolites
Alanine, Glutamate, Aspartate
Glutamine, Asparagine

## Slide 6
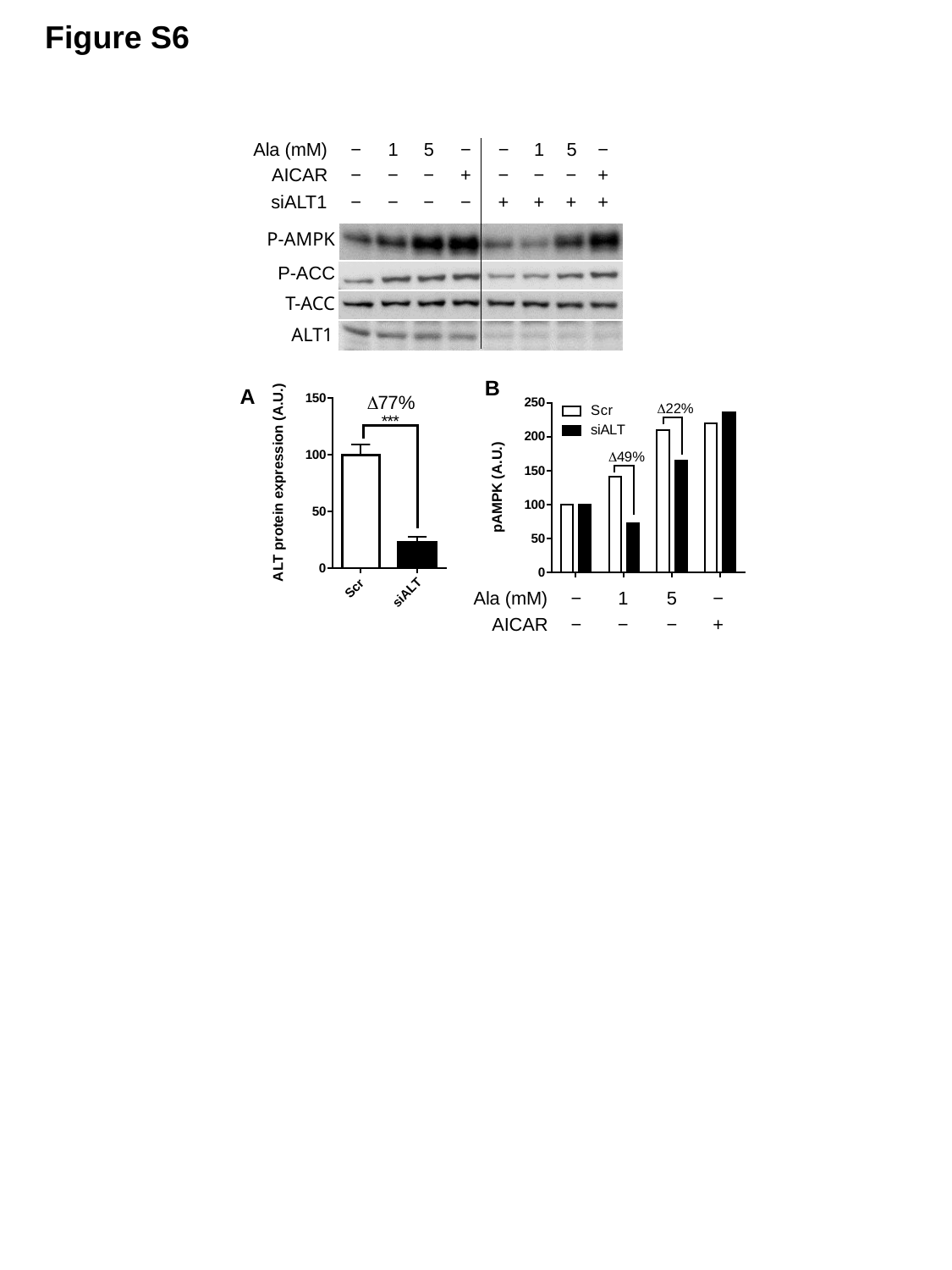

Figure S6
Ala (mM)
−
1
5
−
−
1
5
−
AICAR
−
−
−
+
−
−
−
+
siALT1
−
−
−
−
+
+
+
+
P-AMPK
P-ACC
T-ACC
ALT1
B
A
Ala (mM)
−
1
5
−
AICAR
−
−
−
+
