# Supplementary material for: l-Alanine activates hepatic AMP-activated protein kinase and modulates systemic glucose metabolism
Source: Mol Metab. 2018 Aug 11;17:61–70. doi: 10.1016/j.molmet.2018.08.002 (PMC6197624; doi:10.1016/j.molmet.2018.08.002)
Supplement: Multimedia component 3 [file mmc3.docx]

**Table S1**. Formulation of MDPBS and DMEM used in the present study.

| Composition (g/L) | MDPBS | DMEM (with Pyr) | DMEM (without Pyr) |
| --- | --- | --- | --- |
| CaCl_2_ | - | 0.2 | 0.2 |
| CaCl_2_•2H_2_O | 0.265 | - | - |
| Fe(NO_3_)_3_ • 9H_2_O | - | 0.0001 | 0.0001 |
| MgSO_4_ | 0.09767 | 0.09767 | 0.09767 |
| KCl | 0.2 | 0.4 | 0.4 |
| NaHCO_3_ | 3.7 | 0.37 | 0.37 |
| NaCl | 8 | 6.4 | 6.4 |
| NaH_2_PO_4_ |  | 0.109 | 0.109 |
| Na_2_HPO_4_•7H_2_O | 2.16 |  |  |
| KH_2_PO_4_ | 0.2 | - | - |
| l-Arginine• HCl |  | 0.084 | 0.084 |
| l-Cysteine • 2HCl |  | 0.0626 | 0.0626 |
| l-Glutamine |  | 0.584 | 0.584 |
| Glycine |  | 0.03 | 0.03 |
| l-Histidine• HCl•H_2_O |  | 0.042 | 0.042 |
| l-Isoleucine |  | 0.105 | 0.105 |
| l-Leucine |  | 0.105 | 0.105 |
| l-Lysine• HCl |  | 0.146 | 0.146 |
| l-Methionine |  | 0.03 | 0.03 |
| l-Phenylalanine |  | 0.066 | 0.066 |
| l-Serine |  | 0.042 | 0.042 |
| l-Threonine |  | 0.095 | 0.095 |
| l-Tryptophan |  | 0.016 | 0.016 |
| l-Tyrosine• 2Na• 2H_2_O |  | 0.10379 | 0.10379 |
| Choline Chloride |  | 0.004 | 0.004 |
| Folic Acid |  | 0.004 | 0.004 |
| *myo*-inositol |  | 0.0072 | 0.0072 |
| Niacinamide |  | 0.004 | 0.004 |
| d-Pantothenic Acid• 1/2Ca |  | 0.004 | 0.004 |
| Pyridoxine• HCl |  | 0.004 | 0.004 |
| Riboflavin |  | 0.0004 | 0.0004 |
| Thiamine•HCl |  | 0.004 | 0.004 |
| d-Glucose | 4.5 | 1.0 | 1.0 |
| HEPES |  | - | - |
| Phenol Red•Na | 0.0159 | 0.0159 | 0.0159 |
| Pyruvic Acid•Na |  | 0.11 | - |
